# Potential for Genetic Improvement of the Main Slaughter Yields in Common Carp With *in vivo* Morphological Predictors

**DOI:** 10.3389/fgene.2018.00283

**Published:** 2018-07-30

**Authors:** Martin Prchal, Jérôme Bugeon, Marc Vandeputte, Antti Kause, Alain Vergnet, Jinfeng Zhao, David Gela, Lucie Genestout, Anastasia Bestin, Pierrick Haffray, Martin Kocour

**Affiliations:** ^1^Faculty of Fisheries and Protection of Waters, South Bohemian Research Center of Aquaculture and Biodiversity of Hydrocenoses, University of South Bohemia in České Budějovice, Vodňany, Czechia; ^2^LPGP, INRA, Rennes, France; ^3^GABI, INRA, AgroParisTech, Université Paris-Saclay, Jouy-en-Josas, France; ^4^Ifremer, Palavas-les-Flots, France; ^5^Biometrical Genetics, Natural Resources Institute Finland (Luke), Jokioinen, Finland; ^6^LABOGENA-DNA, Jouy-en-Josas, France; ^7^SYSAAF Section Aquacole, Rennes, France

**Keywords:** heritability estimates, genetic correlations, indirect selection, morphological landmarks, slaughter yields, ultrasound imagery

## Abstract

Common carp is a major aquaculture species worldwide, commonly sold alive but also as processed headless carcass or filets. However, recording of processing yields is impossible on live breeding candidates, and alternatives for genetic improvement are either sib selection based on slaughtered fish, or indirect selection on correlated traits recorded *in vivo*. Morphological predictors that can be measured on live fish and that correlate with real slaughter yields hence remain a possible alternative. To quantify the power of morphological predictors for genetic improvement of yields, we estimated genetic parameters of slaughter yields and various predictors in 3-year-old common carp reared communally under semi-intensive pond conditions. The experimental stock was established by a partial factorial design of 20 dams and 40 sires, and 1553 progenies were assigned to their parents using 12 microsatellites. Slaughter yields were highly heritable (*h*^2^ = 0.46 for headless carcass yield, 0.50 for filet yield) and strongly genetically correlated with each other (*r*_g_ = 0.96). To create morphological predictors, external (phenotypes, 2D digitization) and internal measurements (ultrasound imagery) were recorded and combined by multiple linear regression to predict slaughter yields. The accuracy of the phenotypic prediction was high for headless carcass yield (*R*^2^ = 0.63) and intermediate for filet yield (*R*^2^ = 0.49). Interestingly, heritability of predicted slaughter yields (0.48–0.63) was higher than that of the real yields to predict, and had high genetic correlations with the real yields (*r*_g_ = 0.84–0.88). In addition, both predicted yields were highly phenotypically and genetically correlated with each other (0.95 for both), suggesting that using predicted headless carcass yield in a breeding program would be a good way to also improve filet yield. Besides, two individual predictors (P_1_ and P_2_) included in the prediction models and two simple internal measurements (E4 and E23) exhibited intermediate to high heritability estimates (*h*^2^ = 0.34 – 0.72) and significant genetic correlations to the slaughter yields (*r*_g_ = |0.39 – 0.83|). The results show that there is a solid potential for genetic improvement of slaughter yields by selecting for predictor traits recorded on live breeding candidates of common carp.

## Introduction

Common carp (*Cyprinus carpio* and *C. rubrofuscus*) is highly important freshwater fish species for world aquaculture, with an annual production exceeding 4,000,000 tons (FAO, 2016). Yet, selective breeding programs of carp are less developed than in other aquaculture species (Hulata, 1995; Vandeputte, 2003; Janssen et al., 2017). Crossbreeding of notably inbred strains (Kohlmann et al., 2003, 2005) remains the most used method for genetic improvement of common carp stocks in Europe (Vandeputte, 2003; Nielsen et al., 2010; Janssen et al., 2017). However, the genetic progress is limited only to the first generation, and crossbreeding is not relevant to achieve long term cumulative gains (Nielsen et al., 2010). Selective breeding is more valuable because then the genetic gain is cumulative over multiple generations and a change in the breeding goal is possible over generations (Gjedrem and Baranski, 2009). Nevertheless, selective breeding in common carp is still only emerging and plays a minor role in carp aquaculture (Vandeputte, 2003; Chavanne et al., 2016; Janssen et al., 2017).

Several recent studies have shown a significant additive genetic variation of several performance traits in common carp (Vandeputte et al., 2004, 2008; Kocour et al., 2007; Nielsen et al., 2010; Ninh et al., 2011; Dong et al., 2015; Hu et al., 2017; Prchal et al., 2018) suggesting that important production traits, such as body weight and processing traits, could be genetically improved through selective breeding. Processing traits such as filet yield (filet weight relative to body weight) and carcass yield are more economically valuable traits than body weight itself for species sold processed (Bauer and Schlott, 2009; Kankainen et al., 2016). Processed carp are commonly sold as processed body (headless carcass) or as trimmed filets (Gela et al., 2003; Kocour et al., 2005a, 2007; Bauer and Schlott, 2009).

The use of filet yield in selection programs of fish has been criticized by several authors (Powell et al., 2008; Nguyen et al., 2010a; Gjerde et al., 2012; Van Sang et al., 2012) as in their studies low heritability of filet yield or insignificant response to selection were observed. The conclusion has been that it would be challenging to improve filet weight independently of body weight. A recent simulation study based on field data from three fish species (European sea bass; *Dicentrarchus labrax*, gilthead sea bream; *Sparus aurata* and rainbow trout; *Oncorhynchus mykiss*) indicated that filet yield can be specifically improved in a selection program (Fraslin et al., 2018). Nevertheless, mass selection is not possible in practice as slaughter yields can be only recorded destructively from slaughtered fish. As an alternative, such traits are mostly selected through sib selection or indirect selection on correlated traits recorded *in vivo* (Kause et al., 2007). However, sib selection, where breeding candidates are ranked according to the average performance of their slaughtered sibs, limits the genetic progress by using only genetic variation occurring between-families without exploiting within-family variation (Gjedrem, 2010; Haffray et al., 2013). Such limitation could be overcome by using indirect (non-invasive) selection criteria that can be measured on live breeding candidates, and that would allow exploiting the whole genetic variation related to slaughter yields (Vandeputte et al., 2017). Several studies have reported a possible application of external and internal (ultrasound imagery) morphological measurements predicting filet weight (Cibert et al., 1999; Bosworth et al., 2001; Rutten et al., 2004; Van Sang et al., 2009) or filet yield (Kause et al., 2007; Van Sang et al., 2009; Haffray et al., 2013; Vandeputte et al., 2017), and even their utilization in selective breeding (Kause et al., 2007; Van Sang et al., 2012; Haffray et al., 2013; Vandeputte et al., 2017).

The aim of this study was to (i) determine morphological predictors by external (phenotyping, 2D imaging) and internal measurements (ultrasound imagery) that can be combined by linear regression to predict slaughter yields (headless carcass and filet yields) in common carp, (ii) estimate genetic parameters of slaughter yields and their predictors, (iii) predict and compare the potential genetic gain based on hypothetical mass selection, sib selection and indirect selection based on the predictors of slaughter yields.

## Materials and Methods

### Ethics Statement

The methodological protocol of the current study was approved by the expert committee of the Institutional Animal Care and Use Committee (IACUC) of the University of South Bohemia in České Budějovice (USB), Faculty of Fisheries and Protection of Waters (FFPW) in Vodňany according to the law on the protection of animals against cruelty (Act no. 246/1992 Coll., ref. number 16OZ19179/2016-17214). To enhance animal welfare and decrease suffering during all fish handling, the fish were anesthetized using 2-phenoxyethanol for each live trait recording, and humanely euthanized (humane endpoint) for final recording of slaughter traits. The main author of study owns the certificate (CZ 01704) giving capacity to conduct and manage experiments involving animals according to section 15d paragraph 3 of Act no. 246/1992 Coll.

### Establishment and Rearing of Experimental Stock

In May 2014, the experimental stock was produced at the Genetic Fishery Center of University of South Bohemia (USB) in České Budějovice, Faculty of Fisheries and Protection of Waters (FFPW) in Vodňany, Czech Republic. Amur mirror carp (AM), Vodňany line, recently certified as a new Czech common carp breed (Flajšhans et al., 2015), was chosen as the base population. The AM was used due to its higher genetic diversity (non-published data) compared to other carp breeds available in the Czech Republic that was given by the history of AM establishment. During 1 day, gametes from 20 dams and 40 sires were collected and a partial factorial design with four series of 5 dams and 10 sires in each was used. After fertilization, the eggs from each series were incubated in four separate Zuger jars. Each parental fish was fin-clipped for parentage assignment of the offspring fish. After hatching, the yolk-sac fry from each Zuger jar were transferred and nursed in four separate post-hatching incubators until swimming stage, when the experimental stock was created by pooling equal quantities (estimated volumetrically) of larvae from all four post-hatching incubators. These larvae were released (150,000 larvae. ha^-1^) to the prepared nursery ponds at the Klatovy fish farm. Since then, the families were reared communally in ponds. The families were reared in various pond sizes depending on age of fish and annual period (0.2–4 ha) under semi-intensive pond management based on natural food and supplementary feeding (plant-based pellets altered later with wheat grain) served three times a week. At fish age of 1-year, a random sample of 3000 fish from the best pond (50% survival, mean weight ± SD = 15.8 ± 4.7 g) was anesthetized with 2-phenoxyethanol (0.5 ml per 1 l of water) and individually PIT-tagged and fin-clipped for parentage assignment. After the second growing period and the second overwintering, the fish were harvested and the data were collected for a related study about the genetic potential of overwintering performance in common carp (Prchal et al., 2018). At market size (third growing season) of 1910 g mean weight (October 2016), the fish were harvested and transferred to a storage pond in Vodňany for 3 week-fasting, before final traits recording. This was done to mimic the practice in commercial production, where fasting is used to empty the intestines and to improve the taste and quality of the flesh (Zajic et al., 2013).

### Fish Processing and Final Traits Recording

In November 2016, the fish were transferred to the fish slaughter house of USB FFPW in České Budějovice, Czechia. A total of 1622 individuals were humanely sacrificed by a hit on the head and bled by cutting the gills according to the local rules. These were the fish that had been assigned to a single parental pair based on the microsatellite analysis (see section “Parentage Assignment”). Total length (TL), standard length (SL), body length (BL), head length (HL), body height (BH), and body width (BWI) were measured to the nearest 0.1 mm with an in-house electronic ruler, and body weight (BW) was weighed to the nearest 0.1 g with an electronic scale. For external phenotypic measurements, fish were photographed (left side) using a digital camera (CANON EOS 1000D). Internal measurements were recorded by ultrasound tomography (Hospimedi LC100, 7.5 MHz). The total muscle fat content (% Fat) was recorded using a Fish Fatmeter FM 692 (Distell Ltd., United Kingdom), using calibration option ‘CARP – 1.’ The phenotype for % Fat is expressed as the mean of four repeated measurements (three just above the lateral line from anterior to posterior and one close to the back line in the intermediate part of body) taken on the left side of the fish performed as guided by the manufacturer’s guideline. In addition, selected yield-related biometric indicators were calculated as follows: Fulton’s condition factor: FC = 10^5 ∗^ BW/SL, relative body height: RelBH = BH/SL, and relative head length: RelHL = HL/SL. After biometric recordings, each fish was processed and the following body portions were weighed (to nearest 0.5 g): head, left filet, viscera, gonads (sexed by visual inspection), left filet skin, half carcass, left filet ribs + trimmings, fins, and scales. The weight of slaughter body parts and vertebral axis was created by combining the previous body portions: headless carcass weight [hl-CarssW = left filet + left skin + left ribs and trimmings + half carcass], filet weight with skin [FiletW = (left filet + left filet skin) ^∗^ 2] and vertebral axis weight: [AxisW = half carcass - (left filet + left skin + left ribs and trimmings)]. The percent slaughter yields were calculated as follows: headless carcass yield % [% hl-Carss = (hl-CarssW/BW) ^∗^100], and filet yield [% Fil = (left filet + left skin) ^∗^ 2/BW ^∗^ 100]. In addition, sex effect of analyzed traits was calculated using one-way ANOVA and HSD Tukey test at *p* = 0.05. Finally, to obtain alternative trait definitions to ratio-based traits, the natural logarithm was calculated for the weight of each slaughter body part and regressed on the logarithm of body weight to obtain growth-independent allometry residuals that fix the bias of ratio traits (Gunsett, 1984) and problems connected with estimating of genetic parameters (Gunsett, 1987; Haffray et al., 2013; Vandeputte et al., 2014). Thus, for % headless carcass and % filet yield, the surrogate traits defined as log–log residuals (Logr) are termed as Logr_hl-Carss and Logr_Fil, respectively. To visualize body allometry, logarithm of weight of all body portions mentioned above was regressed on the logarithm of body weight (see Supplementary Figure 1).

### Digitization of 2D Morphometric Landmarks and Ultrasound Tomography

**FIGURE 1:**
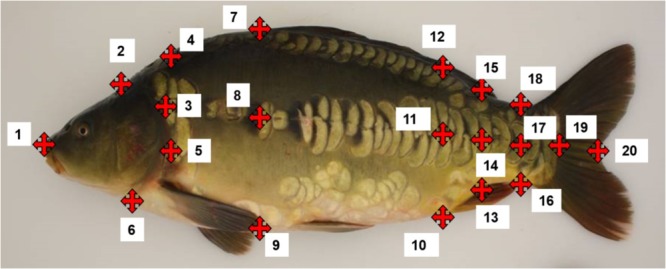
Landmarks placed on each common carp photo. **(1)** Head extremity; **(2)** end of the head beginning of the filet on the back; **(3)** intersection between the back and the vertical of point 4; **(4)** intersection between opercula and lateral line; **(5)** opercula at the maximum length from the landmark 1; **(6)** end of the head beginning of the filet on the ventral part; **(7)** beginning of the dorsal fin; **(8)** intersection between the lateral line and the vertical of landmark 6; **(9)** intersection of the ventral part and the vertical of point; **(10)** beginning of the anal fin; **(11)** intersection between lateral line and vertical of point 9 toward the carp back; **(12)** vertical of point 10 on the back; **(13)** end of anal fin; **(14)** intersection of lateral line and vertical of 12; **(15)** vertical of point 12 on the carp back; **(16)** narrowest point on the caudal peduncle on the back; **(17)** intersection of the lateral line and vertical of 15; **(18)** vertical of point 16 on the ventral part (normally the narrowest point of the caudal peduncle; **(19)** end of the filet (on the skin) on the lateral line; **(20)** end of the caudal fin at the fork.

To quantify the shape of body, head and lateral line, a total of 20 coordinates of morphological points were digitized using ImageJ with the Point Picker plugin (Rueden et al., 2017) that allows storage and retrieval of a collection of landmarks (**Figure 1**). Furthermore, vertical blue lines were added into each image in order to facilitate the manual positioning of landmarks on the surface of the fish. We also checked if the magnification of camera between each working day was unchanged (the difference of pixels of each calibration line was not more/less than 3 pixels out of 2019 pixels. The distances between two landmarks were characterized as *A* (*x*_A_, *y*_A_) and *B* (*x*_B_, *y*_B_) and calculated with the formula: $d=\;\sqrt{{\left(x_{B}-\;x_{A}\right)}^{2}}+\;\sqrt{{\left(y_{B}-\;y_{A}\right)}^{2}}$. These distances were then used to calculate lengths and heights and areas using the Geometry R packages.

The internal measurements were collected using ultrasound imagery (Hospimedi LC1000, 7.5 MHz). Four muscular thicknesses from anterior (E4), intermediate (E5, E8), and posterior (E6) muscles and one internal depth of the body cavity (E23) were measured, at the same position described by Haffray et al. (2013) and Vandeputte et al. (2017).

### 2D Morphology and Prediction Models of Slaughter Yields

The association of the variation in carp morphology to the variation in real processing yields was analyzed using the MorphoJ software (Klingenberg, 2011). This method consists of a Procrustes superimposition of the recorded landmarks used to describe body shape. The log-log residuals slaughter traits, Logr_hl-Carss and Logr_Fil, were used as a surrogate for traditional percent yields and introduced in MorphoJ as a covariates. To quantify the shape variation associated to yield, a regression analysis between Procrustes coordinates and the covariates was performed. The shape changes associated to the covariates were visualized with a wireframe graph. This visualization contributes to identifying relevant morphologic variables to include in a multiple linear regression, for example head area and ventral height.

A multiple linear regression using the reg.best function of the FactoMineR of R software package was performed using the external morphology descriptors, ultrasound measurements and fat meter value as independent variables and the Logr_hl-Carss and Logr_Fil as dependent variables. The best prediction model identification corresponds to those with the highest *R*^2^ and *F*-value. The models were used to calculate the predicted yield values for each fish that are termed as Mod_hl-Carss for headless carcass yield and Mod_Fil for filet yield.

Models were validated by cross validation method using the crossval function of the bootstrap package in R software (Efron and Tibshirani, 1993). Such analysis shows how predictive the equations are on other individuals than the ones that were used to generate the equations. First, the dataset was divided into *K* subsets (here *K* = 20), the analysis is performed on the data of the *K*-1 subsets (training sets) and validated on the data of the remainder of the dataset (validation set). Then the coefficient of determination of the cross validation (*R*^2^CV) was calculated.

### Parentage Assignment

The fin tissues of the 60 parents and 2035 offspring (sampled after the second growing period) were placed into 96 well plates and sent to LABOGENA-DNA, the French laboratory for livestock genotyping (ISO 170025 accredited, Jouy-en-Josas, France). Parentage assignment was based on the analysis of 12 microsatellite loci and performed using the AccurAssign software, applying a maximum-likelihood method (Boichard et al., 2014). The parental pairs retained were chosen using the default thresholds of AccurAssign, i.e., they combined both (i) a difference in log-likelihood between the chosen pair and the second best which was >3 (20 times more likely), and (ii) an average Mendelian transmission probability higher than the highest 99% of 5.000 simulated incorrect trios (dam, sire and offspring).

### Estimation of Genetic Parameters and Expected Genetic Gains

Before genetic analysis, the data quality was checked. The fish for which the total sum of all body portions was greater, or 3% lower than the total body weight were considered as recording errors and excluded from the final analysis. Likewise, a few individuals were also excluded due to aberrant values of external and internal measurements. As a result, 69 fish were excluded and 1553 individuals with a complete set of variables remained in the final analysis. Heritability (*h*^2^), phenotypic and genetic correlations (*r*_p_ and *r*_g_, respectively) were estimated using DMU statistical software (Madsen and Jensen, 2013), with animal mixed model fitted with the restricted maximum likelihood method:

*y* = *X*β + *Z*α + 𝜀

where *y* is the vector of observed phenotypes, *X* and *Z* are appropriate incidence matrices relating phenotypes to vectors β and α. β is the vector of fixed effects (sex with three levels – female, male, unidentified sex) and α is the vector of random additive genetic effects (1613 levels corresponding to all animals – parents and offspring- in the pedigree), and 𝜀 is the vector of random residual effects. The additive (animal) genetic effects were assumed to follow *N*(0, G ⊗ A), with *G* the genetic (co) variance matrix between traits and *A* the numerator relationship matrix relating all animals in the pedigree, while the residual effects were assumed to follow *N*(0, R ⊗ I), *R* the residual (co) variance matrix between traits and *I* an appropriate identity matrix. In the first step, an additional random effect common to dams (non-genetic maternal effect) was included in the model. However, this effect was negligible for all traits, and thus it was not included in the final model.

Heritabilities were estimated using a univariate model, and were calculated as the ratio of additive genetic variance (*V*_A_) divided by the total phenotypic variance (*V*_P_), *h*^2^= *V*_A_/*V*_P_. A model with maximum three traits at a time was used to obtain convergence for genetic correlations. However, when condition factor (FC) was calculated, convergence could not be obtained, and thus the genetic correlations between FC and other traits were obtained from a bivariate analysis of such traits. The likelihood ratio test (LRT) was used for comparing the goodness of fit of two models (including vs. excluding the animal genetic effect). The animal additive genetic effect (and thus the associated heritability estimate) was considered significant when the difference in -2Log-likelihood was higher than the threshold value for *p* < 0.05 of a *χ*^2^ distribution with 1 degree of freedom (Pinheiro and Bates, 2000). Genetic correlation was considered significant if |*r*_g_|-|1.96 × S.E.| was higher than zero (two-tailed hypothesis).

Expected genetic gains (Δ*G*) per generation for filet yield were calculated using the equations of Falconer and MacKay (1996) under a mass (MS), full-sib (FSS) and indirect (IS) selection. The genetic gain under theoretical mass selection based on the lethal criteria was calculated by Δ*G*_MS_ = *i h*^2^ σ*_P_*, where *i* is the selection intensity and *h*^2^ and σ_P_ are the heritability and phenotypic standard deviation of the trait under selection, respectively. The genetic progress of FSS was estimated by $\Delta G_{\mathrm{FSS}}=\frac{i\;\times \;\sigma_{P}\;\times \;h^{2}\;\times \;n\;\times \;r\;\;}{\sqrt{n\left(1+\;\left(n-1\right)\;t\right)\;}}$, where *n* is the number of slaughtered sibs sampled per family (*n* = 10 sibs), *r* is the genetic correlation between sibs (*r* = 0.5 for full sibs) and *t* is the phenotypic intra class correlation (*t* = *rh*^2^). The predicted genetic gain through indirect selection criteria was calculated by Δ*G*_IS_ = *i* × *h*_1_ × *h*_2_ × *r*_g_ × σ_P2_, where Δ*G*_IS_ is the estimated genetic gain on the target trait, *h*_1_ and *h*_2_ are the square roots of heritability of the indirect selection trait (on which selection is applied) and of the target trait, respectively, *r*_g_ is the genetic correlation estimated between the indirect trait and the target trait and σ_P2_ is the phenotypic standard deviation of the target trait. As genetic gains for filets were calculated in log units, the real genetic progress was scaled back to the percent body weight units by multiplying Δ*G* by the real mean filet yield in the present experimental stock. The selection intensities were set up of 10 and 30%, with 10 sibs per family in FSS as the most reasonable values related to a potential carp selection program.

## Results

### Distribution of Families

Out of the 2035 offspring genotyped at the end of the second summer, 1901 (93.4%) could be assigned to a single parental pair, 84 (4.1%) had two possible parent pairs and were considered unassigned, 28 (1.4%) could not be assigned to any parent pair and 23 (1.1%) had DNA quality problems and thus no exploitable genotype. Out of the 1901 uniquely assigned fish, 1622 were still alive at the time of final sampling, and of those 1553 had adequate phenotypes after removal of outliers.

The 1553 fish used in the analysis originated from 197 out of the possible 200 full-sib families. The number of progeny per sire varied from 14 to 79, the average was 39. The number of progeny per dam varied from 25 to 128, the average was 78. The sexes were distributed equally (males – 754, females – 751, unidentified sex – 48).

### Descriptive Statistics of Traits

Mean, standard deviation, differences between sexes (males, females, and unidentified sex) and minimum and maximum values of yield-related traits and slaughter yields are listed in **Table 1**. Sex effect was significant and % Fat, RelBH and both yields were higher for females than for males. Yields of headless carcass (66%) and filets (50%) were higher than usual in common carp, probably due to the specific experimental processing which was different from the commercial one but reflected better the biological characteristics of traits.

**Table 1:** Mean and standard deviation (*SD*) for yield-related traits and percent slaughter yields in males, females and unidentified individuals of common carp.

Trait	Mean ±*SD*	Males^∗^	Females^∗^	Unidentified^∗^	Minimum	Maximum
BW	1910.5 ± 278.9	1899.5^a^ ± 289.4	1923.9^a^ ± 269.8	1873.8^a^ ± 232.2	890.6	2859.5
% Fat	11.56 ± 2.97	10.88^a^ ± 3.06	12.21^b^ ± 2.70	12.10^ab^ ± 3.06	4.10	22.60
FC	3.40 ± 0.32	3.42^a^ ± 0.32	3.38^a^ ± 0.33	3.39^a^ ± 0.25	2.51	5.18
RelBH	0.365 ± 0.023	0.366^a^ ± 0.024	0.364^a^ ± 0.024	0.368^a^ ± 0.020	0.303	0.484
RelHL	0.295 ± 0.013	0.292^a^ ± 0.012	0.297^b^ ± 0.012	0.298^b^ ± 0.013	0.263	0.366
% hl-Carss	66.21 ± 2.19	65.12^a^ ± 2.02	67.27^b^ ± 1.71	67.06^b^ ± 2.90	55.18	72.32
% Fil	49.75 ± 1.95	49.06^a^ ± 1.94	50.41^b^ ± 1.70	50.23^b^ ± 1.92	39.72	55.39

*^∗^Groups with identical alphabetic marker are not significantly different at p < 0.05. BW – body weight, % Fat – percent muscle fat, FC – Fulton’s condition factor, RelBH – relative body height, RelHL – relative head length, % hl-Carss – headless carcass yield, % Fil – filet yield.*

### Body Allometry of Different Body Parts

A positive allometry (regression coefficient >1 in log-log plots) was observed for filet weight, viscera weight and skin weight, showing that heavier fish have proportionally heavier filet, viscera and skin than smaller fish (Supplementary Figure 1). On the contrary, negative allometry was seen for head, vertebral axis, left ribs and trimmings and fins, showing that these parts proportionally decrease in heavier fish. Weights of scales and gonads were hardly linked to body weight. For gonads, there was a clear bimodal distribution, with larger gonads in males than in females (Supplementary Figure 1).

### 2D Morphology and Prediction Models of Slaughter Yields

**FIGURE 2:**
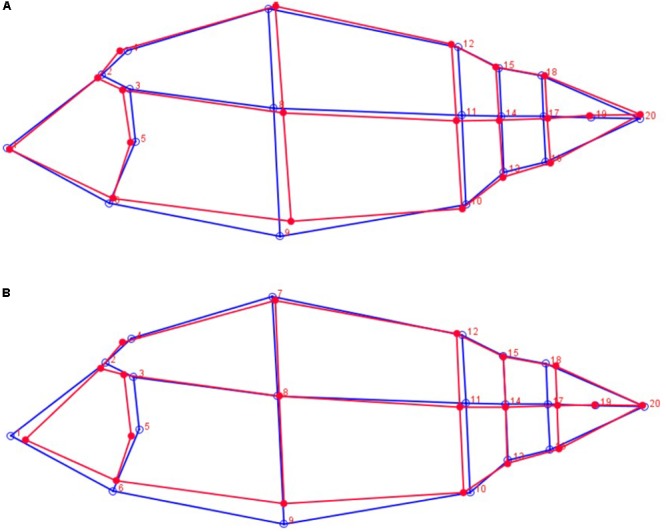
Graphical representation of body morphology for highest values (red lines) and lowest values (blue lines) of Logr_hl-Carss **(A)** and Logr_Fil **(B)**.

A graphical visualization of body morphology associated to low and high yield for Logr_hl-Carss and Logr_Fil is given in **Figure 2**. The main shape differences were observed on the ventral part of the fish and the head. Carp with a high Logr_hl-Carss and Logr_Fil present a lower ventral area especially a lower ventral height under the dorsal fin. Carp with a higher Logr_Fil also have a lower head area with a shorter length between the nose and the operculum. Carp with higher Logr_hl-Carss and Logr_Fil present also a more developed caudal part with a larger caudal peduncle.

The set of best morphological predictors (P_1-5_) included into two prediction equations, and their *R*^2^ and Fisher test values (*F*) are shown in **Table 2**. Logr_hl-Carss could be predicted with a model combining three individual predictors (P_1_, P_2_, P_3_): the ratio of head area to total body area (P_1_), the ratio of abdominal filet thickness to height between the lateral line and the aligned ventral point (P_2_), and the ratio of caudal part area to ventral part area (P_3_). Mod_hl-Carss explains 63% (*R*^2^CV = 0.624) of total phenotypic variance in Logr_hl-Carss. Logr_Fil was best predicted by the model using the same predictors as for Logr_hl-Carss (P_1_, P_2_, P_3_) and in addition body weight (P_4_) and % Fat (P_5_). Mod_Fil explains 49% (*R*^2^CV = 0.489) of total phenotypic variance of Logr_Fil.

**Table 2:** Multiple linear regression models to predict headless carcass (Mod_hl-Carss) and filet yields (Mod_Fil) in common carp including predictors description, *R*^2^, *F* – Fisher test value and prediction equation.

Predicted yield	Predictors	Predictor description	Regression characteristics
Logr_hl-Carss	P_1_	Head area (1-2-4-5-6-1)/total body area (1-2-4-7-12-15-18-19-16-13-10-9-6-1)	*R*^2^ = 0.626, *F* = 866.6, *p* < 0.001, *R*^2^CV = 0.624
	P_2_	Ultrasound E8/height between points 8-9	
	P_3_	Caudal part area (12-15-14-13-10-11-12)/ventral part area (3-8-11-10-9-6-5-3)	Mod_hl-Carss = -0.06–0.37 P_1_ + 6.12 P_2_ + 0.06 P_3_
Logr_Fil	P_1_	Head area (1-2-4-5-6-1)/total body area (1-2-4-7-12-15-18-19-16-13-10-9-6-1)	
	P_2_	Ultrasound E8/height between points 8–9	*R*^2^ = 0.492, *F* = 300.9, *p* < 0.001, *R*^2^CV = 0.489
	P_3_	Caudal part area (12-15-14-13-10-11-12)/ventral part area (3-8-11-10-9-6-5-3)	
	P_4_	Body weight	Mod_Fil = -0.02 – 0.63 P_1_ + 5.30 P_2_ + 0.06 P_3_ -7.84E-06 P_4_ + 0.0007 P_5_
	P_5_	% fat content	

### Heritability Estimates

Heritability estimates of yield-related phenotypes, slaughter yields (Logr) and model-predicted (Mod) slaughter yields are given in **Table 3**. Heritabilities were high for BW and % Fat (0.63 and 0.68, respectively) and maximal (1.00) for traits associated to body shape (FC, RelHL, RelBH).

**Table 3:** Heritability (± standard error) estimates (diagonal) in bold, phenotypic (below the diagonal) and genetic correlations ± standard error (above the diagonal) in common carp for yield-related traits and log-log residuals (Logr) of slaughter yields and models (Mod) to predict slaughter yields.

	BW	% Fat	FC	RelBH	RelHL	Logr_hl-Carss	Logr_Fil	Mod_hl-Carss	Mod_Fil
**BW**	**0.63 ± 0.09**	0.13 ± 0.14	0.45 ± 0.11	0.52 ± 0.10	0.53 ± 0.10	–0.35 ± 0.13	–0.35 ± 0.13	–0.15 ± 0.15	–0.29 ± 0.13
**% Fat**	0.21	**0.68 ± 0.10**	–0.09 ± 0.13	–0.15 ± 0.13	–0.33 ± 0.12	0.25 ± 0.14^1^	0.27 ± 0.14^2^	0.41 ± 0.13^3^	0.56 ± 0.10^4^
**FC**	0.34	0.03	**1.00 ± 0.09**	0.96 ± 0.01	0.78 ± 0.05	–0.10 ± 0.13	–0.17 ± 0.13	–0.15 ± 0.13	–0.25 ± 0.13
**RelBH**	0.40	–0.03	0.88	**1.00 ± 0.09**	0.83 ± 0.04	–0.18 ± 0.13	–0.26 ± 0.13	–0.25 ± 0.13	–0.36 ± 0.12
**RelHL**	0.16	–0.24	0.61	0.64	**1.00 ± 0.10**	–0.47 ± 0.10	–0.53 ± 0.10	–0.47 ± 0.11	–0.64 ± 0.08
**Logr_hl-Carss**	–0.03	0.20	–0.03	–0.04	–0.20	**0.46 ± 0.08**	0.96 ± 0.02	0.88 ± 0.04	0.87 ± 0.04
**Logr_Fil**	–0.02	0.27	–0.02	–0.10	–0.33	0.76	**0.50 ± 0.08**	0.83 ± 0.05	0.84 ± 0.05
**Mod_hl-carss**	0.10	0.27	–0.05	–0.11	–0.27	0.73	0.61	**0.48 ± 0.08**	0.95 ± 0.01
**Mod_Fil**	–0.03	0.43	–0.11	–0.19	–0.42	0.72	0.65	0.95	**0.63 ± 0.09**

*When covariate of body weight to % muscle fat content was used.*

*^*1*^r_*g*_ = 0.40 ± 0.13*

*^*2*^r_*g*_ = 0.42 ± 0.13*

*^*3*^r_*g*_ = 0.55 ± 0.11*

*^*4*^r_*g*_ = 0.68 ± 0.08.*

Logr slaughter yields had higher heritability than the commonly used percentage yields (Logr_hl-Carss, Logr_Fil = 0.46 and 0.50, respectively, vs. 0.36 for percent yields: % hl-Carss, % Fil – Supplementary Table 1). Model yields had a higher heritability than predicted slaughter yields (Mod_hl-Carss, Mod_Fil = 0.48 and 0.63, respectively; **Table 3**).

Heritability estimates of the single predictors used in the models ranged from 0.34 to 0.68 (**Table 4**). Heritabilities of internal measurements were moderate to high (0.34 – 0.72; Supplementary Table 2).

**Table 4:** Heritability (*h*^2^± standard error) of individual predictors (P_1_–P_5_) included in models to predict headless carcass and filet yields and their genetic correlations (*r*_g_) ± standard error with Logr slaughter yields.

	P_1_	P_2_	P_3_	P_4_	P_5_
*h*^2^	**0.34 ± 0.07**	**0.48 ± 0.07**	**0.46 ± 0.08**	**0.63 ± 0.09**	**0.68 ± 0.10**
*r*_g_ Logr_hl-Carss	–0.52 ± 0.12	0.83 ± 0.13	0.29 ± 0.14	–0.35 ± 0.14	0.25 ± 0.14
*r*_g_ Logr_Fil	–0.57 ± 0.11	0.76 ± 0.16	0.34 ± 0.14	–0.35 ± 0.13	0.27 ± 0.14

Heritabilities obtained for allometric log-log residuals of the weights of different body portions to body weight were low for vertebral axis (0.04) and ribs (0.18), which are very prone to measurement errors, and moderate to high (0.31 – 0.62) for the other body parts (Supplementary Table 3). All heritability estimates shown in this study were significantly different from zero (*p* < 0.05).

### Genetic Correlations

Genetic relationship between Logr yields and percent yields was high (*r*_g_ > 0.91; Supplementary Table 1).

The genetic correlations between yield-related phenotypes, Logr and Mod slaughter yields are listed in **Table 3**. Body weight was slightly negatively correlated to both Logr slaughter yields (*r*_g_ = -0.35) and to both predicted slaughter yields (*r*_g_ = -0.15 for Mod_hl-Carss, -0.29 for Mod_Fil). Oppositely, % Fat was positively associated to Logr and Mod slaughter yields (range 0.25–0.56). To ensure that the positive relationships of % Fat with Logr and Mod slaughter yields were not generated by the relation of % Fat with BW, the analysis was also run using BW as a covariate for % Fat. With such a model, the genetic correlations become more positive (*r*_g_ = 0.40–0.68). Body shape traits (FC, RelBH, RelHL) were highly correlated to each other (0.78–0.96) but differed in their relation to slaughter yields. Both FC and RelBH were only slightly negatively and mostly insignificantly correlated to yield traits. Oppositely, RelHL was intermediately negatively associated to Logr and Mod slaughter yields (*r*_g_ = -0.47-0.64).

Logr slaughter yields (Logr_hl-Carss and Logr_hl-Carss) were highly correlated to each other (*r*_g_ = 0.96), similarly as in case of predicted slaughter yields (*r*_g_ = 0.95).

Interestingly real and predicted slaughter yields were highly associated (*r*_g_ = 0.84–0.88), suggesting a good possibility of using predicted yields as indirect selection criterion.

The genetic correlations of the predictors that composed the predictive models with Logr slaughter yields are presented in **Table 4**. P_1_ and P_4_ showed low to moderate negative genetic associations to Logr slaughter yields (-0.35 – -0.57). Oppositely, P_2_ (0.76 – 0.83), P_3_ (0.29 – 0.34), and P_5_ (0.25 – 0.27) were positively correlated to Logr slaughter yields. Hence, individual predictors might be also used in non-invasive genetic improvement of slaughter yields.

The internal measurements E23, E4, E5, E6, E8 achieved negative relationship to the Logr slaughter yields (-0.03 – -0.61), only E8 was in slightly positive relation to Logr yields (Supplementary Table 2). Consequently, simple internal measurements may be useful alternatives for *in vivo* selection for improved yields.

The correlations between Logr body portion yields, BW and % Fat are presented in Supplementary Table 3. The genetic correlation between head (Logr_Head) and left filet (Logr_LFil) was negative (-0.61), showing that fish with smaller head have more filet yield. There was also a positive genetic correlation of % Fat with viscera yield (Logr_Viscera = 0.63), showing that filet fat and viscera (consisting largely of fat) share some common genetic basis. Gonad yield was negatively correlated with left filet yield (*r*_g_ Logr_LFil = -0.49), viscera yield (*r*_g_ Logr_Viscera = -0.40), % Fat (*r*_g_ = -0.46), ribs yield (*r*_g_ Logr_Ribs = -0.65) and fins yield (*r*_g_ Logr_Fins = -0.50) implying a tradeoff of investing in reproduction compared to somatic growth and reserves.

### Expected Genetic Gains

Expected genetic gains using various selection schemes are listed in **Table 5**. Absolute genetic gains for the hypothetical mass selection on real filet yield were 0.70 and 0.46% per generation when 10 and 30% selection intensities were applied, respectively. Genetic gain for FSS with 10 sibs selected per family with the 10 and 30% selection pressure was slightly lower (0.61 and 0.40%) than for mass selection. Estimated genetic gains achieved by indirect selection on the predictor Mod_Fil were 0.66% for 10% selection intensity and 0.43% for 30% which is better than FSS and only slightly lower than direct mass selection on filet yield (which is not possible in practice). Genetic gains ranged from 0.15 to 0.52% for the single predictors used in the models, and from 0.21 to 0.51% for best two internal measurements.

**Table 5:** Expected genetic gain – E.G.G. (in percent body weight units) per generation with two selection intensities (% selected – 10%, 30%) using mass (MS), full sib (FSS), and indirect (IS) selection for filet yield improvement.

Trait selected	Target trait	Type of selection	E.G.G. with 10%	E.G.G. with 30%
Logr_Fil	% Fil	MS	0.70%	0.46%
Logr_Fil	% Fil	FSS	0.61%	0.40%
Mod_Fil	% Fil	IS	0.66%	0.43%
P_1_	% Fil	IS	0.33%	0.22%
P_2_	% Fil	IS	0.52%	0.34%
P_3_	% Fil	IS	0.23%	0.15%
P_4_	% Fil	IS	0.27%	0.18%
P_5_	% Fil	IS	0.22%	0.15%
E23	% Fil	IS	0.51%	0.34%
E4	% Fil	IS	0.31%	0.21%

## Discussion

The present study provided important results relative to the possibility to genetically improve processing yields in common carp: (i) we found high heritability estimates of real and predicted slaughter yields showing a solid potential for their genetic improvement; (ii) high positive genetic correlations were observed between the real and the predicted yields, showing that the latter might be used as non-invasive selection criteria; (iii) expected genetic gain achieved by indirect selection on the predicted yields were higher than those obtained by sib selection that is traditionally applied for improvement of traits needing destructive recording. Thus, we showed that selection of common carp for improved slaughter yields should be feasible, even in a simple breeding program using indirect selection criteria.

### Sex Effect

In this study, sex of the fish had a significant effect on some traits, including slaughter yields. Conversely, BW was found to be independent of sex. However, females had significantly greater relative head length, muscular fat and both yields. This is in accordance with the previous studies performed on common carp in Central European conditions (Kocour et al., 2005a,b, 2007). The explanation is that at market size after the third growing season, female gonads are in younger developmental stage, whereas males are practically mature with fully developed gonads, and thus females have higher slaughter yields. Accordingly, the sex effect was included as a fixed effect in the final genetic model used for estimation of genetic parameters. In later ages, the differences between sexes decrease (Kocour et al., 2005a).

### Genetic Parameters of Yield-Related Traits and Slaughter Yields

Heritability estimates of yield-related traits and body morphology were high and in the upper range when compared to previous studies done on the different batches of common carp (Ankorion et al., 1992; Vandeputte et al., 2004, 2008; Kocour et al., 2007; Nielsen et al., 2010; Dong et al., 2015; Hu et al., 2017) showing a solid potential for genetic improvement of such traits.

The slaughter yields in fish are commonly calculated as a ratio between the given processed body part weight and body weight. However, ratio traits are often biased by growth allometry that is common between body portions and body weight (Gunsett, 1984), and ratios also cause problems when genetic parameters and expected genetic responses are estimated (Gunsett, 1987; Haffray et al., 2013; Vandeputte et al., 2014). On the other hand, such problems might be overcome by calculation of simple residuals (or log-log residuals) between the component traits of a ratio (feed efficiency, slaughter yields) as proposed and applied by Haffray et al. (2012) and Vandeputte et al. (2014, 2017), and in this study. In the present study, heritabilities of slaughter yields expressed as log–log residuals (Logr) were higher (0.46–0.50) than the heritabilities for percent slaughter yields (0.36 for both slaughter traits). The latter are more in line with the previous study using also percent slaughter yields (*h*^2^ = 0.28–0.36; Kocour et al., 2007). However, yields as residuals and percent yields were highly genetically correlated showing that the both variables explain the same trait similarly as described by Vandeputte et al. (2017). Therefore, residuals are more valuable surrogates for slaughter yields both due to their higher inheritance and the potential biases of ratio traits mentioned above.

We observed a strong genetic correlation between Logr_hl-Carss and Logr_Fil (0.96 ± 0.02). Likewise, Kocour et al. (2007) estimated high but lower genetic relationship between slaughter yields in common carp (0.79 ± 0.13). A similar genetic association between yields was found in rainbow trout (0.97 ± 0.01; Haffray et al., 2013), and European sea bass (0.79 ± 0.20; Vandeputte et al., 2017). Our study confirms that, similar to rainbow trout and sea bass, headless carcass yield (faster processing, less technical errors) might be proposed as a reliable surrogate for filet yield, especially when sib selection (evaluated on slaughtered sibs) is applied for genetic improvement of carp yields.

Harvest body weight and Logr slaughter yields were slightly negatively genetically correlated (*r*_g_ = -0.35). On the contrary, a high positive genetic correlations of body weight and slaughter yields (0.73–0.74) were found previously in common carp (Kocour et al., 2007). However, in this case slaughter yields were expressed as percent ratios and might have been effected by positive growth allometry. Therefore, comparison of these two studies is not relevant. On the other hand, even when Logr type of traits are used, such correlations are not consistent among other fish species, with zero genetic correlations observed in European sea bass (Vandeputte et al., 2017) and slightly negative correlations observed in rainbow trout (Haffray et al., 2012). This points to the fact that such correlations are probably breed and species specific and modified by biological and/or genetic phenomena between growth and slaughter yields across fish species. In our scenario, body weight should be integrated in a selection index with slaughter yields, to avoid a negative impact on growth when selecting for slaughter yields.

Positive genetic correlations were observed between % Fat and Logr slaughter yields (*r*_g_ = 0.25–0.27). A strong genetic relationship of % Fat to percent yields (0.66–0.76) was reported earlier in common carp (Kocour et al., 2007). So, selection for improved yields would indirectly lead to a slight increase of fat in the muscle. However, an excessive increase of muscle fat level without a change in the feeding strategy might lead to an unfavorable decrease of beneficial omega-3 polyunsaturated fatty acids (n-3 PUFAs) in the muscle (Nguyen et al., 2010b). Thus, selection program for increased percent yield may worsen the quality of final product. A selection program focused on increased edible parts yields should minimize risk of this phenomena using appropriate measures, e.g., by simultaneously controlling lipid deposition (Bugeon et al., 2010; Nguyen et al., 2010b; Janhunen et al., 2017).

The traits related to body shape, FC and RelBH, were slightly negatively related to slaughter yields implying that selection for improved yields in long term may change the general body shape. This is visible also in **Figure 2** where body morphology for the fish with the highest slaughter yields is represented by a more prolonged body shape. A similar relationship of body shape to % yields was observed in common carp (Kocour et al., 2007) and other fishes (Navarro et al., 2009; Haffray et al., 2012; Van Sang et al., 2012). On the other hand, due to its very high heritability, body shape itself might be changed quite simply in common carp by direct selection, as reported by Prchal et al. (2018) and proved in a selection experiment by Ankorion et al. (1992).

The relative head length (RelHL) was moderately negatively correlated to both yields (-0.47 – -0.53) implying that selection for lower RelHL could be an indirect selection criterion for increased yields in common carp. This is in agreement with Kocour et al. (2007), where even stronger negative genetic correlations were observed. Moreover, both percent or Logr head yield were also negatively associated to slaughter yields in other fish species (Rutten et al., 2005; Kause et al., 2007; Saillant et al., 2009; Haffray et al., 2012; Vandeputte et al., 2017). However, there are gills in head, main respiratory organ of fish, so selection for lower RelHL in a long term selection program might lead to functional damage of respiration, adaptation or osmoregulation capacities (Haffray et al., 2012; Fraslin et al., 2018) and this could affect general fish performance and fitness. Moreover, selection for lower RelHL has to be considered with caution and in any case integrated in a global selection index due to a high positive genetic correlation between RelHL and BW (*r*_g_ = 0.53) as well as RelBH (*r*_g_ = 0.83). Uncontrolled selection for a smaller relative head length may thus lead to a limitation of gains in growth and faster change to an oblong-like body shape that may be less favorable for some carp consumer buying whole fish on the traditional market.

### Genetic and Phenotypic Parameters of Predicted Slaughter Yields

Phenotypic correlations between Logr and Mod yields were moderately high (0.73 for headless carcass, 0.65 for filet yields). The accuracy of phenotypic prediction was high for Logr_hl-Carss (*R*^2^ = 0.63), and intermediate for Logr_Fil (*R*^2^ = 0.49). Such prediction of slaughter yields, combining external and internal measurements, was recently performed on rainbow trout (Haffray et al., 2013) and European seabass (Vandeputte et al., 2017). Our phenotypic predictions of yields were more accurate compared to these studies (*R*^2^ = 0.38 for headless carcass yield in Haffray et al., 2013, *R*^2^ = 0.02 – 0.18 for filet and 0.27 – 0.41 for carcass yield in Vandeputte et al., 2017). Hence, slaughter yields can be effectively predicted on live breeding candidates in common carp. Remarkably, Mod_hl-Carss is easier to construct in comparison with Mod_Fil (3 predictors vs. 5 predictors), and it has higher phenotypic prediction accuracy and strong phenotypic and genetic correlations (0.95 for both) to Mod_Fil. Thus, Logr headless carcass is recommended as a trait to be predicted to select for improved filet yields. This is also supported by its favorably lower negative genetic relation to the body weight and lower positive association to % Fat. In addition, Mod yields achieved high heritability (0.48–0.63), higher than Logr yields (*h*^2^ = 0.46–0.50), and also higher when compared to other studies in which inheritance of predicted yields were estimated (Van Sang et al., 2012; Haffray et al., 2013; Vandeputte et al., 2017). This is important as it shows a good possibility of using Mod yields as an indirect selection criterion, further supported by high genetic correlations between Logr and Mod yields (0.84–0.88). It must be stressed that our results were obtained from data recorded on Amur mirror carp in semi-intensive pond conditions and at fish market size specific to Central and Eastern Europe. Validation of the predictors would be necessary before their utilization on other carp breeds, strains, lines, and size categories. Still, many of our conclusions are in line with those drawn from the previous studies in rainbow trout (Kause et al., 2007; Haffray et al., 2012, 2013), European sea bass (Vandeputte et al., 2017) and a previous small-scale study on common carp (Kocour et al., 2007), and thus our results are expected to have a reasonable level of generality.

Heritability estimates of individual predictors, that were included in the prediction models, were moderate for ratio predictors (0.34–0.48) and high for BW (P_4_) and % Fat (P_5_) (0.63–0.68). In the recent studies (Haffray et al., 2013; Vandeputte et al., 2017), *h*^2^ for predictors from which the models were constructed ranged from 0.06 to 0.54 for various simple and combined predictors. Besides, P_1_ and P_2_ predictors were moderately to highly genetically correlated with the Logr yields. P_1_ was defined as a ratio between head area to total body area (2D measurements) with negative association to yields. So, selection on lower value of P_1_ would lead to higher yields as smaller head is related to higher yields (*r*_g_ = -0.52–0.57) similar to RelHL discussed above. P_2_ was the ratio between ultrasound measurement of abdominal thickness (E8) and external belly height measured between landmarks 8 and 9 in 2D, and was highly positively associated to yields (*r*_g_ = 0.76–0.83). A similar relation occurs in rainbow trout (Haffray et al., 2013) with the ultrasound measurements ratio of E8 to E23. Thus, P_2_ could be an even more suitable indirect selection criterion in genetic improvement of slaughter yields.

Although the added value of external morphology combined with internal measurements is interesting, the time needed for trait recording and the accuracy of prediction are more in favor of simple ultrasound measurements. Accordingly, rapid internal measurements (especially E4 and E23) might be used as alternative indirect criteria in accordance to their high heritability and intermediately high genetic correlations to yields (see Supplementary Table 2). Furthermore, 3D collection of external body landmarks could accelerate digitization of potentially relevant morphological predictors as proposed by Haffray et al. (2013). Thus, 2D and 3D collection of morphological landmarks and their power to predict yields should be under further research.

### Expected Genetic Gain

Based on the expected genetic gain calculations, full-sib selection (FSS) would produce slightly lower genetic improvement than hypothetical mass selection (MS) applied on filet yields in both selection intensities. Still, sib selection might be effectively applied in genetic improvement of common carp yields. In addition, FSS method could be practically performed on real headless carcass yield, which is easier to be measured and less prone to measurement errors than filet yield, but has a simultaneous favorable effect on filet yields due to the high genetic correlation between both (0.96). On the other hand, sib selection utilizes only between-family genetic variation without exploiting genetic variation within families (Gjedrem, 2010; Haffray et al., 2013).

On the other hand, indirect selection using Mod filet yields (or the simpler Mod_hl-Carss) recorded *in vivo* could overcome limitations from sib selection mentioned above and give an even better response compared to FSS (expected genetic gain was 0.43–0.66% for indirect filet yield improvement). Alternatively, simple internal measurements (E4 and E23) or individual predictors P_1_ and P_2_ might be used as traits for indirect genetic improvement of yields (0.21–0.52%) as it was also suggested by Haffray et al. (2013) and Vandeputte et al. (2017).

Nevertheless, it must be stressed that using Logr yields values that were used for derivation of best predictors and Mod yields might slightly overestimate the potential genetic gains. This was visible when simulation selection analysis was run in accordance with Fraslin et al. (2018) (data not shown). Such bias could be eliminated by linear index theory developed to improve selection gain on ratio traits (Lin, 1980; Lin and Aggrey, 2013), and optimized to improve filet weight/waste weight ratio or filet weight/body weight ratio in fish species (Fraslin et al., 2018). However, it is unclear how linear index theory could be connected to external predictors of yields, as the theory uses absolute values of body portions (weights) and not relative values (yields). Hence, this issue should be under further research.

## Conclusion

In the present study, model-predicted slaughter yields in common carp were highly heritable and strongly genetically associated to highly hereditary real yields, expressed as log-log residuals. The results show potential for genetic improvement of processing yields through selective breeding, also by using *in vivo* morphological predictors. In addition, both real and predicted headless carcass yield might be used as an efficient surrogate (faster processing, easier to predict) for filet yield improvement through sib or indirect selection. Besides, two internal ultrasound measurements and two individual predictors could be also alternatively used as traits for indirect selection in genetic improvement of slaughter yields in common carp. As predictors are combining several sources of information, further information on the resulting breeding accuracies and realized genetic gains would be valuable in the future to quantify the expected progress. Furthermore, validation of best predictors would be necessary before their transfer to other carp breeds, strains, lines, and size categories.

## Data Accessibility

The dataset underlying our findings is fully available in the public data repository (OSF: https://osf.io/vfnbs/).

## Author Contributions

MP, DG, and MK shared on establishing and on-growing the experimental stock, PIT tagging, and fin clipping the fish. PH and MP provided the methodology and equipment. MP, JB, MV, AV, JZ, DG, AB, and MK shared on final trait recordings. JZ digitized the morphological points. AK introduced MP to the quantitative genetic analysis. JB carried out the phenotypic prediction of slaughter yields. LG performed the DNA extractions and parentage assignment. MP and MV estimated the genetic parameters. All authors contributed to drafting the manuscript and approved the final version.

## Conflict of Interest Statement

The authors declare that the research was conducted in the absence of any commercial or financial relationships that could be construed as a potential conflict of interest. The handling Editor declared a past co-authorship with several of the authors MP, MV, AB, PH, and MK.
