# Supplementary material for: Potential for Genetic Improvement of the Main Slaughter Yields in Common Carp With in vivo Morphological Predictors
Source: Front Genet. 2018 Jul 30;9:283. doi: 10.3389/fgene.2018.00283 (PMC6078046; doi:10.3389/fgene.2018.00283)
Supplement: Supplementary file 1 [file Table_1.DOCX]

**Supplementary Table S1:** Heritability estimates (± S.E.), phenotypic and genetic correlations (± S.E.) in common carp for percent and Logr slaughter yields

|  | **% hl-Carss** | **% Fill** | **Logr_hl-Carss** | **Logr_Fill** |
| --- | --- | --- | --- | --- |
| **% hl-Carss** | **0.36 ± 0.08** | 0.96 ± 0.02 | 0.94 ± 0.02 | 0.90 ± 0.03 |
| **% Fill** | 0.78 | **0.36 ± 0.08** | 0.87 ± 0.04 | 0.91 ± 0.03 |
| **Logr_hl-Carss** | 0.96 | 0.70 | **0.46 ± 0.08** | 0.96 ± 0.02 |
| **Logr_Fill** | 0.73 | 0.92 | 0.76 | **0.50 ± 0.08** |
