# Supplementary material for: Potential for Genetic Improvement of the Main Slaughter Yields in Common Carp With in vivo Morphological Predictors
Source: Front Genet. 2018 Jul 30;9:283. doi: 10.3389/fgene.2018.00283 (PMC6078046; doi:10.3389/fgene.2018.00283)
Supplement: Supplementary file 2 [file Table_2.DOCX]

**Supplementary Table S2:** Heritability (± S.E.) of internal measurements and their genetic correlations (± S.E.) with the Logr slaughter yields

|  | **E23** | **E4** | **E5** | **E6** | **E8** |
| --- | --- | --- | --- | --- | --- |
| **Heritability** | **0.72 ± 0.08** | **0.56 ± 0.09** | **0.43 ± 0.09** | **0.34 ± 0.07** | **0.42 ± 0.08** |
| **Logr_hl-Carss** | -0.57 ± 0.11 | -0.39 ± 0.14 | -0.32 ± 0.14 | -0.11 ± 0.16 | 0.29 ± 0.14 |
| **Logr_Fill** | -0.61 ± 0.10 | -0.42 ± 0.13 | -0.29 ± 0.15 | -0.03 ± 0.16 | 0.25 ± 0.15 |
