# Supplementary material for: Potential for Genetic Improvement of the Main Slaughter Yields in Common Carp With in vivo Morphological Predictors
Source: Front Genet. 2018 Jul 30;9:283. doi: 10.3389/fgene.2018.00283 (PMC6078046; doi:10.3389/fgene.2018.00283)
Supplement: Supplementary file 3 [file Table_3.DOCX]

|  | **BW** | **% Fat** | **Logr_Head** | **Logr_LFill** | **Logr_Viscera** | **Logr_Gonads** | **Logr_LSkin** | **Logr_Axis** | **Logr_LRibs** | **Logr_Fins** | **Logr_Scales** |
| --- | --- | --- | --- | --- | --- | --- | --- | --- | --- | --- | --- |
| **BW** | **0.63 ± 0.09** | 0.13 ± 0.14 | 0.49 ± 0.13 | -0.45 ± 0.11 | -0.06 ± 0.15 | 0.26 ± 0.14 | 0.29 ± 0.14 | 0.25 ± 0.25 | -0.20 ± 0.17 | 0.08 ± 0.16 | -0.24 ±0.14 |
| **% Fat** | 0.21 | **0.68 ± 0.10** | -0.45 ± 0.13 | 0.21 ± 0.14 | 0.63 ± 0.10 | -0.46 ± 0.12 | 0.20 ± 0.14 | -0.54 ± 0.24 | 0.39 ± 0.15 | 0.36 ± 0.14 | 0.30 ± 0.14 |
| **Logr_Head** | 0.04 | -0.28 | **0.31 ± 0.06** | -0.61 ± 0.10 | -0.19 ± 0.16 | 0.05 ± 0.16 | 0.30 ± 0.15 | 0.70 ± 0.18 | -0.06 ± 0.18 | 0.02 ± 0.16 | -0.45 ± 0.13 |
| **Logr_LFill** | -0.08 | 0.20 | -0.39 | **0.62 ± 0.09** | -0.12 ± 0.15 | -0.49 ± 0.12 | -0.43 ± 0.12 | -0.41 ± 0.25 | 0.26 ± 0.16 | -0.07 ± 0.16 | 0.37 ± 0.13 |
| **Logr_Viscera** | 0.08 | 0.41 | -0.10 | -0.04 | **0.44 ± 0.08** | -0.40 ± 0.13 | 0.08 ± 0.15 | -0.47 ± 0.23 | 0.22 ± 0.17 | 0.31 ± 0.15 | 0.26 ± 0.14 |
| **Logr_Gonads** | 0.12 | -0.16 | -0.03 | -0.38 | -0.39 | **0.54 ± 0.09** | -0.11 ± 0.15 | 0.07 ± 0.27 | -0.65 ± 0.11 | -0.50 ± 0.13 | -0.17 ± 0.15 |
| **Logr_Skin** | 0.01 | 0.18 | 0.17 | -0.28 | 0.04 | -0.07 | **0.56 ± 0.09** | 0.23 ± 0.26 | 0.10 ± 0.17 | 0.23 ± 0.15 | -0.65 ± 0.09 |
| **Logr_Axis** | 0.01 | -0.12 | -0.32 | -0.17 | -0.05 | -0.10 | -0.21 | **0.04 ± 0.02** | 0.05 ± 0.30 | 0.10 ± 0.26 | -0.67 ± 0.23 |
| **Logr_Ribs** | -0.04 | 0.08 | 0.07 | 0.15 | 0.01 | -0.33 | -0.01 | -0.14 | **0.18 ± 0.05** | 0.42 ± 0.15 | 0.07 ± 0.17 |
| **Logr_Fins** | -0.01 | -0.05 | 0.23 | -0.15 | -0.07 | -0.07 | 0.08 | -0.10 | 0.09 | **0.36 ± 0.07** | 0.13 ± 0.16 |
| **Logr_Scales** | -0.10 | 0.06 | -0.17 | 0.12 | 0.12 | -0.07 | -0.39 | -0.01 | 0.01 | 0.01 | **0.57 ± 0.09** |

**Supplementary Table S3:** Heritability estimates (± S.E.), phenotypic and genetic correlations (± S.E.) in common carp for body weight, % Fat and all Logr body portions.
