# Supplementary figures and images for: Potential for Genetic Improvement of the Main Slaughter Yields in Common Carp With in vivo Morphological Predictors
Source: Front Genet. 2018 Jul 30;9:283. doi: 10.3389/fgene.2018.00283 (PMC6078046; doi:10.3389/fgene.2018.00283)

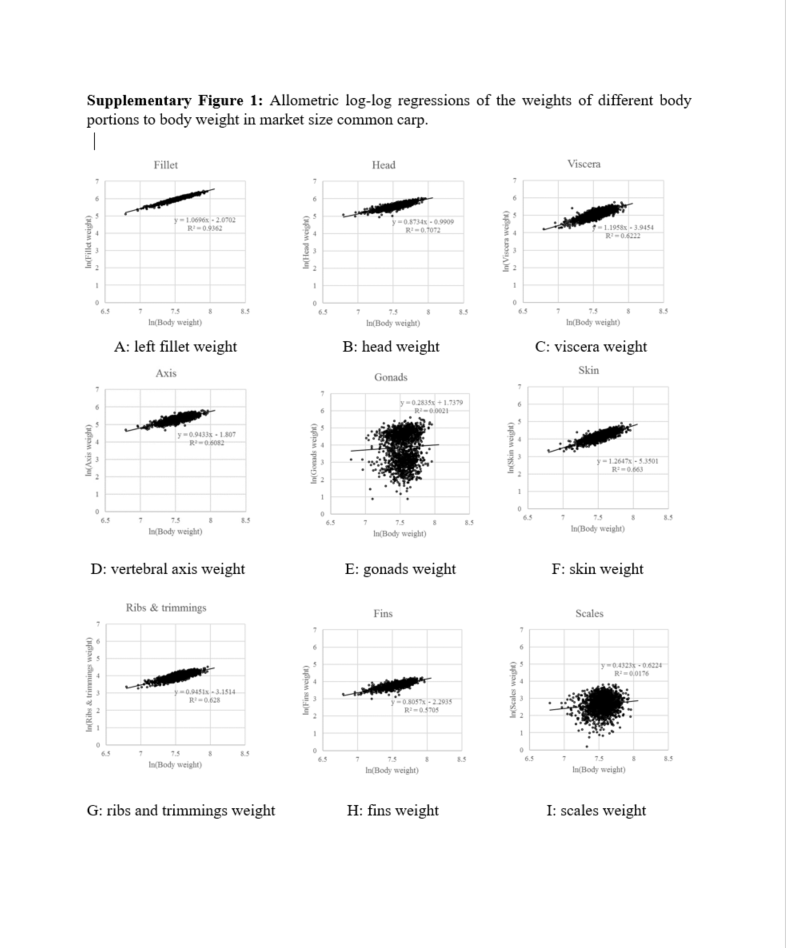

Supplement: Supplementary file 4 [file Image_1.TIF]
